# The Effect of HLA Polymorphism on Immune Response to SARS‐CoV‐2 Vaccination Within an Infection‐Naïve, Vulnerable Population With End‐Stage Renal Disease

**DOI:** 10.1111/tan.70076

**Published:** 2025-02-24

**Authors:** Fiona Erskine, Katrina Spensley, Maria Prendecki, Eva Santos, Arthi Anand, Danny Altmann, Michelle Willicombe

**Affiliations:** ^1^ Imperial College London Department of Surgery and Cancer London UK; ^2^ Imperial College Healthcare NHS Trust London UK; ^3^ NHS North West London Pathology London UK

## Abstract

HLA genes exhibit a high degree of polymorphism, contributing to genetic variability known to influence immune responses to infection. Here we investigate associations between HLA polymorphism and serological and T‐lymphocyte responses to the BNT162b2 and ChAdOx1 SARS‐CoV‐2 vaccines within a population receiving maintenance haemodialysis (HD) for End‐Stage Renal Disease (ESRD). Our primary objective was to identify HLA alleles associated with diminished serological and T‐cellular responsiveness to vaccination. As a secondary objective, the associations between HLA type and COVID‐19 disease outcomes were investigated using an independent ESRD cohort (*n* = 327). This aimed to determine if the alleles associated with poor vaccine response were also linked to unfavourable infection outcomes. In the main study, serum from 225 SARS‐CoV‐2 infection‐naïve patients was HLA‐typed using high‐resolution Next Generation Sequencing, and serological titres were analysed for the presence of SARS‐CoV‐2 spike glycoprotein‐specific antibodies after two doses of vaccination. A subset of patients (*n* = 33) was also tested for a T‐lymphocyte response. Overall, 89% (*n* = 200) of patients seroconverted, but only 18% (*n* = 6) of the cellular response subgroup had a positive T‐lymphocyte response. The HLA class II alleles DPB1*104:01, DRB1*04:03 and DRB1*14:04 and HLA class I alleles B*08:01 and B*18:01 were found to significantly correlate with seronegativity, and DQB1*06:01 correlated with serological responsiveness. We were unable to analyse the effect of HLA on disease outcome and T‐lymphocyte response due to sample size limitations. Our results suggest pathways for further research and begin to elucidate the relationship between HLA polymorphism and immune responses in the vulnerable ESRD population.

## 1 Introduction

Host genetic profiles have proven to be a valuable component of studies into COVID‐19 disease susceptibility [1, 2]. Among loci implicated in SARS‐CoV‐2 vulnerability are a number of sites impacting innate and adaptive immune mechanisms, including polymorphisms in the HLA system [3, 4, 5, 6]. In addition to factors like age, BMI, sex and diabetes [7, 8, 9, 10], polymorphisms that alter peptide‐binding affinities in the HLA system have been shown to drive variability in adaptive immune responses to COVID‐19 infections [4, 5, 11]. Furthermore, the capacity of HLA polymorphism to influence immunogenicity has been demonstrated across a range of vaccinations [12, 13, 14], and it has been well established that neutralising antibody levels are a key predictor of COVID‐19 immune protection [15, 16].

Both the BNT162b2 mRNA (Pfizer‐BioNTech) and ChAdOx1 nCoV‐19 (Oxford‐AstraZeneca) SARS‐CoV‐2 vaccines show efficacy in reducing hospitalisations, severe disease manifestations, and mortality across international populations [17, 18, 19]. However, a number of patients remain unresponsive to vaccination [20, 21].

This investigation centred on patients undergoing haemodialysis (HD) for ESRD (End Stage Renal Disease). These patients constitute a uniquely vulnerable cohort with respect to COVID‐19, owing to the established immunosuppressive effects of ESRD and HD [22, 23]. Additionally, their hospital‐based haemodialysis appointments during the height of the pandemic augmented their viral exposure. While several studies have explored the link between HLA type and SARS‐CoV‐2 infection response [24, 25, 26, 27, 28], there is less robust data concerning the effect of HLA polymorphism on BNT162b2 and ChAdOx1 vaccine responses [29], and minimal investigation on vaccine responses within a vulnerable population such as that included in this study.

This paper examines the effect of HLA type on serological and T‐lymphocyte responses following two doses of either BNT162b2 or ChAdOx1 SARS‐CoV‐2 vaccines in an ESRD population and assesses the influence of HLA on COVID‐19 disease outcomes prior to vaccination. The principal aim of the study was to identify HLA alleles associated with a low immune response, thereby providing a comprehensive understanding of how HLA polymorphism influences vaccinated patients' susceptibility to SARS‐CoV‐2.

## 2 Materials and Methods

### 2.1 Patient Cohort

**FIGURE 1:**
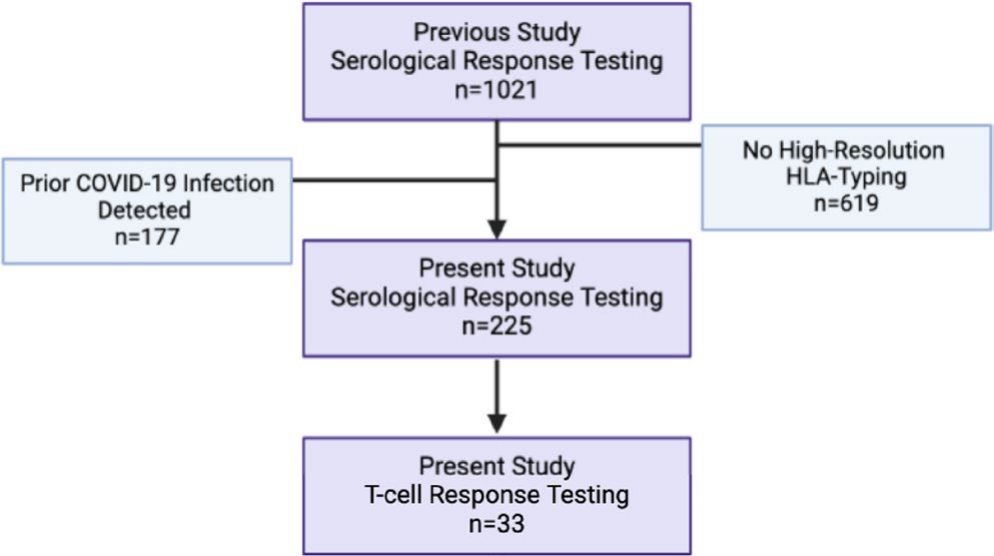
Study flow diagram. Sourcing of haemodialysis (HD) patients for serological (*n* = 225) and T‐lymphocyte (*n* = 33) response testing. This study is a continuation of a previous study by our group [28] (*n* = 1021) conducted in 2022, which assessed the efficacies of SARS‐CoV‐2 vaccines within the HD cohort from the Imperial College Healthcare NHS Trust. Purple boxes represent the study sourcing flow, and blue boxes represent groups that were excluded from the investigation.

Our group has previously described serological responses after two SARS‐CoV‐2 vaccines in 1021 haemodialysis patients [20]. All participants were receiving routine haemodialysis on March 1st, 2020, across nine facilities belonging to Imperial College Renal and Transplant Centre. From these 1021 patients, we identified 225 patients for this study who had been high‐resolution HLA‐typed using Next‐Generation Sequencing and who were infection‐naïve, as defined below (Figure 1). Two standard doses of either the BNT162b2 or ChAdOx1 vaccines were administered within the study period of March–July 2021. Patients were sourced from the Imperial College Healthcare NHS Trust and monitored post‐second vaccine (V2) until the 30th of November 2021. A subgroup of 33 (15% of present study patients) randomly selected patients underwent analysis of T‐lymphocyte responses to spike antigen following vaccination. The study was approved by the Health Research Authority, Research Ethics Committee (20/WA/0123), and the relevant clinical data were obtained from patient electronic health records.

### 2.2 SARS‐CoV‐2 Serological Testing

Serological samples were collected at a median of 42 days (IQR: 28–56 days) following vaccination. Serum was tested for spike (anti‐S) antibodies using the qualitative and semi‐quantitative Abbott Architect SARS‐CoV‐2 IgG Quant II chemiluminescent immunoassay (CMIA), in accordance with the manufacturer's instructions. A result of < 7.1 BAU/ml was used as the manufacturer's cutoff to define a negative or negligible antibody response. Patients were classified as seropositive if they reported a positive anti‐S titre after their 2nd vaccination. Abbott reports high sensitivity (92·7% (95% CI 90.2–94.8)) and specificity (99·9% (99.4%–100%)) of the assay.

### 2.3 SARS‐CoV‐2 Cellular Response Testing

T‐cell SARS‐CoV‐2 responses were detected using the T‐SPOT Discovery SARS‐CoV‐2 (Oxford Immunotec), according to the manufacturer's instructions. Cellular immune responses to SARS‐CoV‐2 spike peptide pools (S1 protein and S2 protein) were detected, and a positive PHA (phytohemagglutinin) and negative controls were included. Blood samples were taken from patients during haemodialysis sessions, and peripheral blood mononuclear cells (PBMCs) were isolated and treated with T‐Cell XtendTM (Oxford Immunotec) where appropriate. 250,000 PBMCs were plated into individual wells of the T‐SPOT Discovery SARS‐CoV‐2 plate. Cells were incubated, and interferon‐γ (IFN‐γ) producing T‐cells were detected with an automated plate reader (Autoimmun Diagnostika). As previously described [30], unvaccinated participants with no prior SARS‐CoV‐2 infection were used to define T‐cell non‐response. An IFN‐gamma response of < 40 SFU/10^6^ PBMC following spike antigen stimulation was defined as non‐response.

### 2.4 Identifying Prior SARS‐CoV‐2 Infection

Prior SARS‐CoV‐2 infection was defined by the presence of any one of the three following characteristics that were detected:

1. Positive viral detection from nasopharyngeal swab specimens using reverse‐transcriptase polymerase chain reaction (RT‐PCR) assays.
2. Positive anti‐NP serology at any time point.
3. Positive anti‐S serology before vaccination.

All haemodialysis patients underwent nasopharyngeal swabbing screening every week during the study period, and serological screening was conducted every 3 months from June 2020.

### 2.5 HLA‐Typing

ESRD patients were routinely HLA‐typed for the purposes of transplantation; this cohort includes those awaiting transplantation and those who have returned to dialysis following transplant failure. High‐resolution typing was performed at the Histocompatibility and Immunogenetics Laboratory within Imperial College NHS Healthcare Trust and was carried out with GenDx MX6‐1 HLA typing kits. HLA‐A, ‐B, ‐C, ‐DRB1, ‐DQB1 and ‐DPB1 alleles were recorded. Sequencing reactions were performed using the Illumina iSeq platform, and results were analysed on the GenDx NGS engine.

### 2.6 Statistical Analysis

#### 2.6.1 Analysis of Demographic Data

Demographic data from the patient cohort were analysed using Mann–Whitney U testing (continuous data) and Fisher's Exact Test (discrete data). Any attribute deemed to significantly (*p* < 0.05) affect serological/cellular response was included in the logistic regression model. Significant attributes and well‐established predictors of serological/cellular responsiveness were included as forced variables in the logistic regression model.

#### 2.6.2 Building the Logistic Regression Model and Subdivision of Participants

Logistic regression models were run with serological or cellular response as the outcome and the forced variables as predictors. If a patient's observed serological/cellular response did not match the model's prediction, they were “mismatched” whereas if the observed response matched the prediction, the individual was “matched”. The HLA allelic frequencies of the mismatched and matched patients were compared using proportional assessment.

#### 2.6.3 Proportional Assessments for the Identification of Alleles of Interest

Proportional assessments were conducted on each allele in the study to identify “alleles of interest”. Allelic frequencies from matched patients were compared with mismatched and any HLA allele that had a cohort‐wide frequency of less than three was excluded from further analysis. Fisher's Exact Test was used for proportional assessment if the cohort‐wide frequency of the allele was five or fewer and Chi‐Squared testing was used for frequencies greater than five. Alleles were considered of interest if the associated *p*‐values were less than 0.1. This less conventional threshold was chosen to prioritise exploratory inclusivity and reduce the risk of omitting potentially significant alleles, recognising that subsequent multivariate modelling would address any alleles that did not meet stricter significance criteria. Collinearity analysis was conducted using Pearson correlation coefficients and corresponding *p*‐values, where alleles with values of less than 0.05 were considered significantly collinear.

#### 2.6.4 Multivariate Analysis

Multivariate models were used to determine if alleles of interest were independent predictors of serological or cellular responses. The alleles of interest were included in multivariate logistic regression models in addition to demographic predictors. From the coefficient estimates calculated in the multivariate analysis, odds ratios (OR) and 95% confidence intervals (CI) were evaluated, and any allele whose 95% CI did not cross the null effect point at OR = 1 and whose *p*‐value from multivariate analysis was less than 0.05 was considered a significant predictor of serological or cellular response. All statistical analyses were conducted using RStudio (Rstudio Team, 2023).

### 2.7 Infection Response Analysis

This arm of the study was open to patients on dialysis within the West London Renal Transplant Centre on March 1st, 2020. 327 ESRD patients were included and followed up until fully vaccinated. 104 patients were HLA‐typed using high resolution typing as described above; the remaining 223 patients were HLA‐typed with intermediate resolution typing. There were no common patients between the infection and vaccination response analyses. Typing was carried out using the LABtype HLA‐SSO typing kits, according to the manufacturer's instructions. Data analysis was performed using the HLA Fusion Software.

From the intermediate resolution data, the most likely high‐resolution HLA type was imputed using the HLA Matchmaker Four‐digit Allele Converter Program (v01) (www.epitopes.net/downloads.html), which generates high‐resolution allele information. Input includes HLA‐A, ‐B and ‐DR at low/medium resolution and 1 of 4 racial/ethnic groups. Output is the most common high‐resolution HLA‐A, ‐B, ‐C, ‐DR and ‐DQ haplotypes for the patient's race or ethnicity. The HLA profiles of individuals who never reported a positive SARS‐CoV‐2 test during the test period (23rd March 2020 to 24th April 2021) were compared with the profiles of patients who tested positive. An additional HLA analysis was also conducted between those who experienced severe COVID‐19 infections (required hospitalisation or died) and those with mild infections (no hospitalisation required). Statistical analysis for infection response was carried out as described for serological and T‐lymphocyte responses to vaccination. To address potential confounding from non‐genetic factors, the logistic regression model included demographic covariates such as age, sex, ethnicity and dialysis subgroup.

## 3 Results

### 3.1 Cohort Clinical and Demographic Data

Demographic data was analysed for the 225 infection‐naïve patients (Table 1); 200 (88.9%) had a positive antibody response following vaccination, constituting the seroconversion cohort, where seropositivity refers to IgG titres of Anti‐S > 7.1 BAU/ml. 25 (11.1%) patients failed to seroconvert.

**TABLE 1:** Demographic information of study participants.

Characteristics		Seroconversion *n* = 200 (%)	Failed to seroconvert *n* = 25 (%)	*p*
Age	Years—median (IQR)	58.4 (48.2–65.3)	57.1 (48.4–62.3)	0.67
Diabetes	Yes	88 (44)	12 (48)	0.83
	No	112 (56)	13 (52)	0.83
ESRD cause	APKD	9 (5)	1 (4)	1
	Urological	16 (8)	2 (8)	1
	GN	44 (22)	8 (32)	0.31
	DM	72 (36)	8 (32)	0.83
	Other	15 (8)	4 (16)	0.24
	Unknown	44 (22)	2 (8)	0.12
Ethnicity	Caucasian	59 (30)	11 (44)	0.17
	Black	32 (16)	5 (20)	0.57
	Indoasian	72 (36)	5 (20)	0.12
	Other	37 (18)	4 (16)	1
Immunosuppression	Yes	31 (16)	17 (68)	**< 0.0001**
	No	169 (84)	8 (32)	**< 0.0001**
Interval between V1 and V2	Days—Median (IQR)	61 (37–70)	62 (39–67)	1
Interval between V2 and sera sample	Days—Median (IQR)	42 (30–62)	35 (28–50)	0.67
On the transplant list	Yes	69 (35)	6 (24)	0.37
	No	131 (65)	19 (76)	0.37
Sex	Female	131 (65)	18 (72)	0.65
	Male	69 (35)	7 (28)	0.65
Time at ESRD	Years—Median (IQR)	2.0 (0.6–5.2)	5.6 (1.9–12.9)	0.33
Vaccine type	ChAdOx1 (AstraZeneca)	101 (51)	19 (76)	**0.02**
	BNT162b2 (Pfizer)	99 (49)	6 (24)	**0.02**

*Note:* Clinical characteristics of the study participants (*n* = 225), attributes found to be significantly associated with seroconversion were vaccine type (*p* = 0.02) and immunosuppressive medication status (*p* < 0.0001). Proportional assessment was conducted with Mann U Whitney testing or Fisher's Exact Test where appropriate and reported *p*‐values correspond with the results of these tests.

Abbreviations: ESRD, end‐stage renal disease; V1, vaccine dose 1; V2, vaccine dose 2.

Overall, 120 (53%) patients were administered the AstraZeneca ChAdOx1 vaccine and 105 (47%) received the Pfizer BNT162b2 vaccine. While the seroconversion group possessed roughly equal numbers of ChAdOx1 and BNT162b2 vaccinees (*n* = 101; 51% and *n* = 99; 49%, respectively), the non‐seroconversion cohort had significantly more ChAdOx1‐vaccinated patients (76%, *n* = 19, *p* = 0.02). Subscribing to immunosuppressive medication was also significantly associated with non‐seroconversion. 21% (*n* = 48) of the total cohort and 16% (*n* = 31) of those who seroconverted were immunosuppressed compared with 68% (*n* = 17) of non‐seroconverts (*p* < 0.0001).

Other clinical characteristics considered were not found to be significant determinants of serological response by proportional assessment within this cohort (age, diabetes status, cause of ESRD, ethnicity, time interval between V1 and V2, interval between V2 and sera sample, transplant list status, sex and the time at ESRD).

When designing the serological and cellular regression models, demographic attributes were included if they were found to be significant in the cohort proportional assessments (vaccine type and immunosuppression status) or if they had been well established in the literature to significantly affect antibody or cellular response to the SARS‐CoV‐2 vaccines (age, sex, ethnicity, time at ESRD and interval between V2 and sera sample) [31, 32, 33].

Participants were subdivided into four groups based on the results of the logistic regression model's predicted response (informed by significant demographic predictors immunosuppression status, vaccine type, age, sex, ethnicity, ESRD type and V2‐sera interval) and their observed seroconversion or cellular response in clinic (Table 2). Group 1 functioned as a comparator (control) for Group 2, which was composed of patients whose responses could not be explained by the model built on the demographic predictors discussed above. Allelic frequency differences were investigated between Groups 1 and 2 to ascertain which alleles correlated with non‐responsiveness. No analysis was conducted between Groups 3 and 4 due to low subject numbers.

**TABLE 2:** Subdivision of study participants for HLA frequency analysis.

	Group 1	Group 2	Group 3	Group 4
Predicted response	Yes	Yes	No	No
Observed response	Yes	No	No	Yes
Patients (*n* = 225)—antibody testing	*n* = 197	*n* = 17	*n* = 7	*n* = 4
Patients (*n* = 33)—cellular testing	*n* = 5	*n* = 0	*n* = 27	*n* = 1

*Note:* Protocol for group subdivision for further analysis of serological and T‐lymphocyte response post‐vaccination. Observed Response refers to the patient's seroconversion/cellular status while Predicted Response refers to the regression model's seroconversion/cellular response prediction for the patient. Group 1 was the comparator group for Group 2. The majority (*n* = 197) of serological‐response testing patients were in Group 1, while the majority of T‐lymphocyte‐response testing patients (*n* = 27) were in Group 3.

### 3.2 Associations Between HLA Alleles and Seroconversion

Comparative analysis of HLA allele frequencies between Groups 1 (*n* = 197) and 2 (*n* = 17) by proportional assessment revealed 10 alleles of interest (Table 3). These alleles were A*03:01 (*p* = 0.05), A*26:01 (*p* = 0.09), B*08:01 (*p* = 0.04), B*18:01 (*p* = 0.004), B*50:01 (*p* = 0.07), C*12:03 (*p* = 0.07), DPB1*104:01 (*p* = 0.03), DQB1*06:01 (*p* = 0.08), DRB1*04:03 (*p* = 0.03) and DRB1*14:04 (*p* = 0.07).

**TABLE 3:** Proportional assessment of allelic frequency differences between groups.

Allele	Group 1 Freq.	Group 2 Freq.	Significance (*p*)
A*03:01	*n* = 23	*n* = 5	0.053
A*26:01	*n* = 11	*n* = 3	0.088
B*08:01	*n* = 21	*n* = 5	0.040
B*18:01	*n* = 11	*n* = 5	0.004
B*50:01	*n* = 10	*n* = 3	0.072
C*12:03	*n* = 17	*n* = 6	0.003
DPB1*104:01	*n* = 7	*n* = 3	0.035
DQB1*06:01	*n* = 33	*n* = 0	0.081
DRB1*04:03	*n* = 6	*n* = 3	0.026
DRB1*14:04	*n* = 10	*n* = 3	0.072

*Note:* The results of proportional assessments (Chi‐Squared or Fisher's Exact Test, where appropriate) between groups 1 (*n* = 197) and 2 (*n* = 17) for serological‐response testing. 10 alleles (hereafter referred to as “alleles of interest”) that demonstrated a significant difference between groups were identified using *p* < 0.1 and are shown in the table above.

**FIGURE 2:**
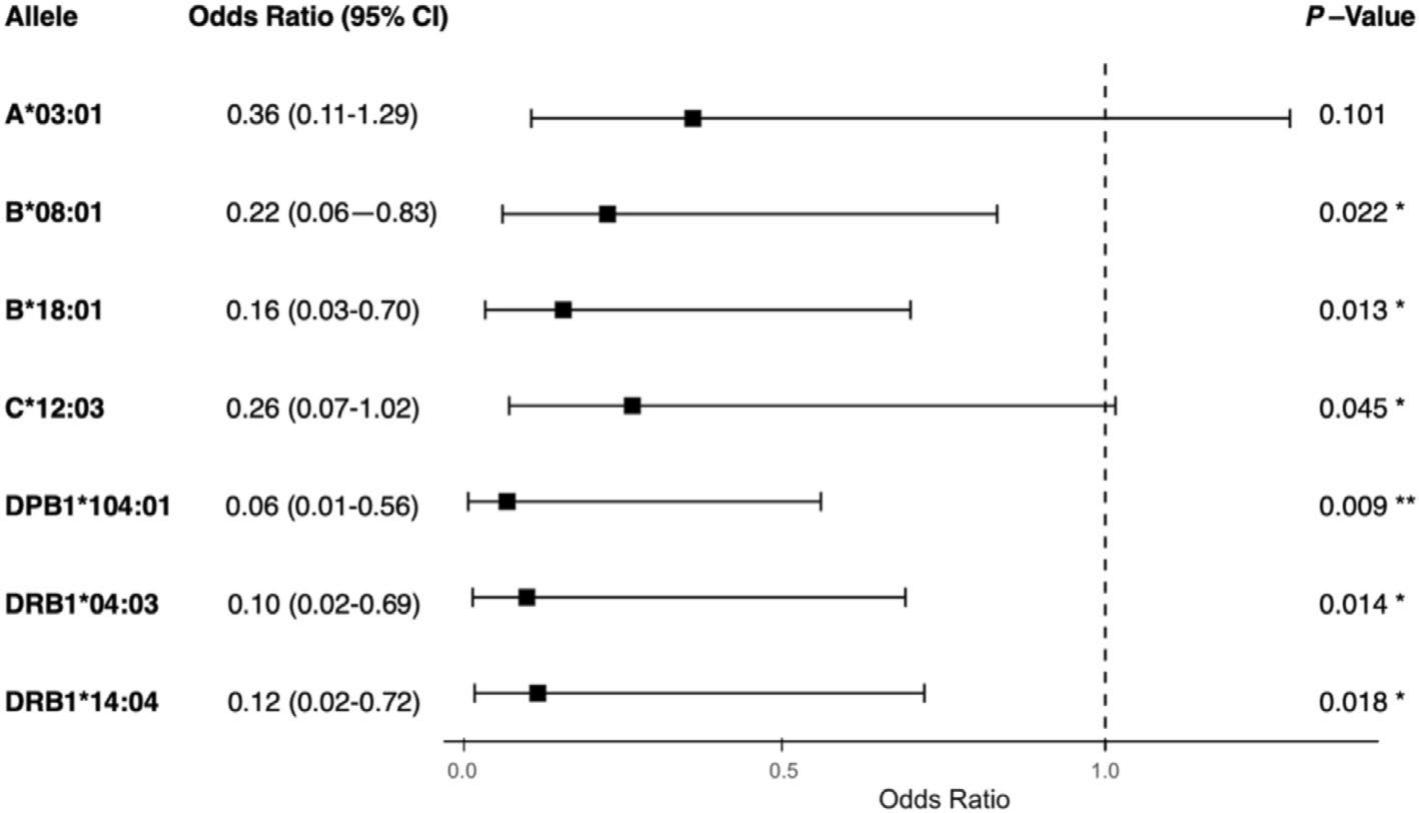
Alleles of interest associated with serological non‐responsiveness. A graphical representation of the odds ratios and 95% confidence intervals of the alleles of interest suspected to be predictors of antibody non‐responsiveness, and associated p‐values from the *Z* statistic calculated from the logistic regression models. The null effect point is represented by a dashed line at OR = 1. B*08:01, B*18:01, DPB1*104:01, DRB1*04:03 and DRB1*14:04 were significant. Error bars represent 95% confidence intervals for odds ratios. (.) *p* < 0.1, (*) *p* < 0.05, (**) *p* < 0.01.

A collinearity analysis was performed on the alleles of interest to investigate linked polymorphisms (Figure S1 and Table S1). From the analysis, three pairs of significantly associated alleles were identified; these were A*26:01–B*08:01 (*p* < 0.001), B*18:01–C*12:03 (*p* < 0.00001) and B*50:01–DPB1*104:01 (*p* < 0.001). A*26:01 and B*50:01 only appeared in the profiles of Group 2 patients alongside their linked alleles and so were excluded from the analysis. Both B*18:01 and C*12:03 appeared in Group 2 HLA profiles as a pair and independently, so a note was made of the collinearity, and both were included in the multivariate modelling (Figure 2).

Ultimately, DPB1*104:01 (OR = 0.06, 95% CI: 0.01–0.56, *p* = 0.009), DRB1*04:03 (OR = 0.10, 95% CI: 0.02–0.69, *p* = 0.014), DRB1*14:04 (OR = 0.12, 95% CI: 0.02–0.72, *p* = 0.018), HLA‐B*08:01 (OR = 0.22, 95% CI: 0.06–0.83, *p* = 0.022) and B*18:01 (OR = 0.16, 95% CI: 0.03–0.70, *p* = 0.013) were found by multivariate modelling to be significant predictors of antibody non‐responsiveness post‐vaccination.

Less significant was the predictive capacity of the A*03:01 (OR = 0.36, 95% CI:0.11–1.29, *p* = 0.101) and C*12:03 (OR = 0.26, 95% CI: 0.07–1.02, *p* = 0.045) alleles. Although indicated to be of interest by proportional assessment, the evidence is not strong enough to rule out the possibility of no effect.

Further analysis was not possible with DQB1*06:01 since it was only present in the HLA profiles of Group 1 patients. This rendered logistic regression inapplicable since it is unable to calculate instances where a variable perfectly predicts an outcome due to the complete separation problem [34].

### 3.3 Associations Between HLA Alleles and Cellular Response

As with serological response, a regression model for cellular response was designed to account for demographic covariate factors including immunosuppression status, vaccine type, ethnicity, age, sex, time diagnosed with ESRD, and the time interval between V2 and serology samples.

33 of the 225 study patients (15%) were chosen at random and tested for T‐lymphocyte IFN‐γ responses after exposure to the spike antigen post‐V2. Only 18% (*n* = 6) of the infection‐naïve patients had a positive T cell response after vaccination, and 82% (*n* = 27) had no detectable response. Additionally, no patients were allocated to Group 2 (Table 2), preventing any comparative analysis from being completed between groups.

### 3.4 Associations Between HLA Alleles and Infection Response

The relationships between imputed HLA type and susceptibility to COVID‐19 infection were analysed using an independent cohort of 327 ESRD patients, whose demographics are summarised in Table S2. A logistic regression model for infection response was designed to account for the demographic covariate factors age, sex, ethnicity and dialysis subgroup (dialysis non‐waitlist, dialysis waitlist, pandemic transplant). The infection response analysis was two‐pronged and included a comparison of the HLA profiles of those who never tested positive for SARS‐CoV‐2 (*n* = 176) vs. those with a positive test (*n* = 151), and a comparison between the HLA profiles of severe (*n* = 56) vs. mild (*n* = 95) COVID‐19 disease presentations.

After proportional assessment, five HLA class I alleles were identified to be of interest from the imputed dataset for increased risk of infection; these were A*01:01, A*26:01, B*08:01, B*18:01, C*07:01. After regression modelling, only A*26:01 was significant (OR = 0.16, 95% CI: 0.04–0.47, *p* = 0.003). In the analysis of mild vs. severe presentations of COVID‐19, alleles A*68:01 and DRB1*04:03 were of interest for the risk of severe infection and were exclusively present in the profiles of Group 2 patients. However, sample size constraints prevented the regression analysis necessary to determine their statistical significance.

## 4 Discussion

This investigation aimed to identify HLA alleles correlating with serological and T‐lymphocyte responses following two doses of SARS‐CoV‐2 vaccination within the vulnerable ESRD patient cohort. Of 225 infection‐naïve patients who were assessed for seroconversion, 89% (*n* = 200) had an antibody response and 11% (*n* = 25) failed to seroconvert. T‐lymphocyte responsiveness to the spike antigen was tested in 15% (*n* = 33) of this study cohort and 18% (*n* = 6) had positive responses. Three HLA class II alleles (DPB1*104:01, DRB1*04:03 and DRB1*14:04) and two HLA class I alleles (B*08:01 and B*18:01) were significantly associated with serological non‐responsiveness to vaccination. Analysis of T‐lymphocyte response to vaccination was limited by the small sample size, and HLA type imputation limited our confidence in the analysis of infection responses; however, our data suggest interesting avenues for further analysis.

Among the three HLA class II alleles identified as significant predictors of serological non‐responsiveness in this study, DRB1*14:04 and DRB1*04:03 have also been previously identified for being over‐represented in COVID‐19 patients with severe disease and those requiring hospitalisation [35, 36, 37]. Additionally, the infection response analysis conducted in parallel to this investigation found DRB1*04:03 to be an allele of interest for severe infection. Furthermore, while several studies have identified associations between increased serological response following COVID‐19 vaccine and DRB1 alleles [28, 38, 39] others have failed to demonstrate a significant association between DRB1 alleles and failure to seroconvert [28, 40], which is in opposition to the allelic associations found in this investigation.

Following proportional assessment, DQB1*06:01 was the only allele significantly under‐represented in the HLA profiles of seronegative patients. Prior to this study, the role of DQB1*06 alleles in enhancing seroconversion was well documented. Mentzer et al. [41, 42, 43] established the association between DQB1*06 and higher levels of S antibody following ChAdOx1 vaccination, and additional studies have identified this allele group as protective against severe infection. Furthermore, Xie et al. [38] conducted an in‐depth investigation into the DQB1*06 allele subtype and found that DQB1*06:01,02,03,04 and09 all correspond to enhanced seroconversion levels, and that DQB1*06:04 was the most significant. In this investigation, only DQB1*06:01 was found to be a statistically significant enhancer of antibody response post vaccination, likely due to a low cohort‐wide frequency of the other related alleles. Additionally, several DRB1 alleles previously identified as being protective against seronegativity, including DRB1*15:01, which is in linkage disequilibrium with DQB1*06:01 and DRB1*13:02 [28, 38], also showed reduced expression in the Group 2 cohort but did not pass the significance threshold. It is also worth noting that DRB1*15:01 and DQB1*06:02 only appeared once in the Group 2 profiles, in the same patient, and that both alleles appeared many times in the seroconversion group. Nevertheless, our results support the trend of resistance to seronegativity with this allelic subtype.

In addition to HLA class II alleles, this study found the HLA class I alleles B*08:01 and B*18:01 to be associated with seronegativity. This was an unexpected result given that there is poor evidence to support a pathway linking HLA class I to antibody response [44]. A possible explanation for this occurrence lies in the strong and complex linkage disequilibrium patterns of HLA genes. In 21 of 25 appearances in the cohort, B*08:01 was found to be co‐inherited with both DRB1*03:01 and DQB1*02:01, suggesting that the class II alleles in the haplotype may be influencing the resulting seronegativity. This is further supported by two previously published studies that identified a significant correlation between the HLA‐A*01:01–B*08:01–C*07:01–DRB1*03:01–DQB1*02:01 haplotype and low serological response to SARS‐CoV‐2 vaccination and severe disease manifestation [45, 46]. Moreover, Crocchiolo et al. found the haplotype A*24:02–B*18:01–C*07:01–DRB1*11:04 to be associated with a low serological response to vaccination [29], which may provide an explanation for the significance of B*18:01 detected in this study.

Curiously, both of these alleles also appeared to be of interest, but not significant, in the infection response analysis using an independent ESRD cohort, as did A*01:01, C*07:01 and A*26:01, which was shown to be highly linked to B*08:01. These data provide further support for the idea that the summative effect of these haplotypes may have a complex connection with the systemic responses to the virus, elucidating which would require fine‐mapping and functional genomic analyses beyond the scope of this paper. Nevertheless, the present study adds to the growing body of evidence suggesting a class I association with SARS‐CoV‐2 serological response, highlighting the need for further investigation into this area [29, 45].

A major assumption of this investigation was that instances in which the logistic regression model failed to accurately predict the observed serological response were wholly attributed to the effects of the HLA profile. In reality, while HLA variations are very likely to affect serological response [47], a number of other genetic, environmental, or ESRD disease factors could limit a patient's ability to seroconvert [48]. Indeed, only ~50%–75% of haemodialysis patients vaccinated against HBV, influenza, and diphtheria develop antibodies compared with ~90% in healthy controls. Of clinical relevance is that these vaccines are all protein antigen‐based, as are the SARS‐CoV‐2 vaccines described in this paper. HD patients do not show reduced seroconversion levels to polysaccharide‐antigen vaccines [23], indicating a fundamental T‐cell disturbance may be a primary reason for seronegativity, an explanation that is supported by the low frequency of T‐lymphocyte responses to the spike antigen seen in this investigation.

Many limitations encountered in this analysis can be attributed to small allelic sample sizes, a consequence of the polymorphic nature of HLA genes. Only the most common alleles in the population achieved frequencies of greater than 50, limiting the statistical power of the assessment. This also offers an explanation as to why alleles with similar hypervariable regions and those previously linked to seronegativity or infection incidence in other studies were not found to be significant in our analysis. In addition, although this investigation covers a broad spectrum of ethnicities, ages and other demographics to provide an accurate model of the British public, caution should be taken when extrapolating these findings to the general population given the patients' subscription to immunosuppressive medications and haemodialysis treatment necessitated by the ESRD condition. Additionally, certain HLA types known to be linked to ESRD, such as HLA‐B*08, may be overrepresented in this cohort compared to a non‐diseased population [49, 50, 51]. The haemodialysis patients included in this study may also not fully represent the overall disease cohort, as only those being considered for transplantation were included. Finally, it is worth noting that the study population consists of patients who survived the first wave of SARS‐CoV‐2 in 2020 and are thus more likely to harbour protective HLA phenotypes.

Furthermore, non‐genetic factors, such as protective behaviours and exposure risks, which are inherently difficult to quantify, likely influenced the infection susceptibility analysis, which was a secondary objective of this study. Although discrepancies in protective behaviours were somewhat mitigated by the shared necessity of in‐person haemodialysis appointments during the height of the pandemic, which exposed patients to similar levels of risk, future studies with larger cohorts and more detailed behavioural data will be essential to further validate the genetic associations identified here.

Further analysis using a larger ESRD patient population would also likely address many of the sample size and power limitations encountered in this study and could also uncover additional alleles that have an effect on seroconversion but were under‐represented in this study. Additionally, while the sample sizes were also insufficient to analyse HLA differences between patients who failed to respond to vaccination as predicted (Group 3) and those who responded despite not being predicted to do so (Group 4), future research exploring these differences could yield insights into protective factors and unexpected serological responsiveness.

Moreover, a detailed analysis of T‐lymphocyte responses distinguishing between helper and cytotoxic T‐cells across a broader patient population could uncover the specific immune mechanisms that affect responses to SARS‐CoV‐2 vaccination. This would be especially relevant given the emerging research highlighting the importance of the cytotoxic T‐lymphocyte response to vaccination in providing lasting immunity, suggesting that it may be as crucial as serological responses in serving as a correlate of protection [52, 53].

Finally, despite the limitations, this study is one of the largest and most comprehensive analyses of the association between HLA polymorphism and SARS‐CoV‐2 vaccine response, as well as one of the only studies to investigate this topic within a vulnerable population. Findings here may be useful for identifying patients who require additional risk mitigation measures, such as booster vaccines or adherence to social distancing protocols. Additionally, given the study's diverse racial representation and the strong link between HLA type and ethnicity [54, 55, 56, 57], the results may also guide ethnicity considerations surrounding vaccination and risk mitigation strategies for other vulnerable populations not routinely HLA typed, such as those with heart disease and diabetes [58]. Overall, these results have the potential to give significant insight not only into the immune mechanisms at play in immunosuppressed patients but also to expand our understanding of the intricacies of the HLA system and viral immune response.

## 5 Conclusions

Our findings suggest a role of specific HLA polymorphisms in the determination of serological response to SARS‐CoV‐2 vaccination in infection‐naïve patients with ESRD. Further research with greater study participants is necessary to elucidate the relationship between HLA polymorphism and T‐lymphocyte response, a critical area of investigation for patients with ESRD due to their established T‐cell dysfunction. A refined understanding of immune variation with regard to vaccination is critical for the protection of vulnerable patients, and our results support the idea that the identification of immunogenetic determinants of risk will be the key to accomplishing such a goal.

## Author Contributions

F.E. wrote the manuscript text and prepared the tables and figures. K.S., M.P., E.S., A.A., D.A. edited and provided guidance for the methodology and main text. M.W. conceptualised and initiated the project. All authors reviewed the manuscript.

## Conflicts of Interest

Michelle Willicombe has received support to use the T‐SPOT Discovery SARS‐CoV‐2 by Oxford Immunotec and speaker fees from AstraZeneca. Danny Altmann has received honoraria for consulting work with Pfizer, AstraZeneca, Oxford Immunotec and Novavax. He sits on the Scientific Advisory Board of BerlinCures and is a trustee of Long Covid Support. The other authors declare no conflicts of interest.

## Supporting information


**Data S1.** Supporting Information.

## Data Availability

The data that support the findings of this study are available on request from the corresponding author. The data are not publicly available due to privacy or ethical restrictions.
