# Supplementary material for: The Effect of HLA Polymorphism on Immune Response to SARS‐CoV‐2 Vaccination Within an Infection‐Naïve, Vulnerable Population With End‐Stage Renal Disease
Source: HLA. 2025 Feb 24;105(2):e70076. doi: 10.1111/tan.70076 (PMC11848999; doi:10.1111/tan.70076)
Supplement: Supplementary file 1 — Data S1. Supporting Information. [file TAN-105-e70076-s001.docx]

**Supplementary Figures and Tables**

**Figure S1 – Correlation Plot for Alleles of Interest**


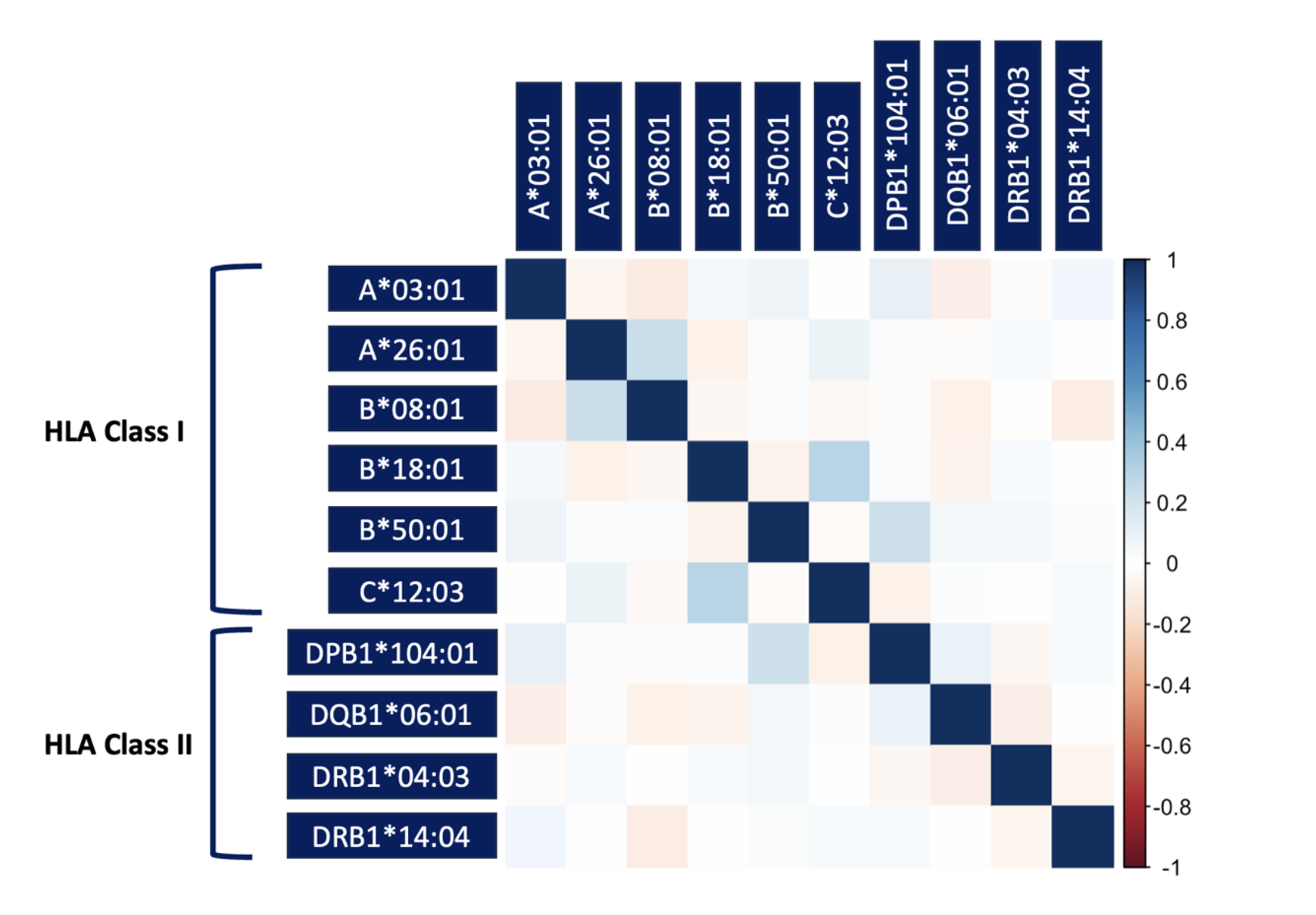


Figure S1- Correlation heatmap of alleles of interests for serological testing post-vaccination. As indicated, blue squares show alleles that are positively correlated, or more likely to be inherited together and red squares indicate alleles that are negatively correlated and more likely not be inherited together. Three pairs of alleles showed possible collinearity, these were A*26:01~B*08:01, B*18:01~C*12:03, and B*50:01~DPB1*104:01.

**Table S1 – Association Collinearity P-Values for Alleles of Interest**

Table S1- P values for Collinearity. This table corresponds with the heatmap in Figure S1, Pearson’s correlation coefficients from the collinearity analysis were used to calculate p-values for correlation between each allele of interest. P-values less than 0.05 denoted significantly related alleles and these are marked in green. Three pairs of allele showed significant collinearity, these were A*26:01~B*08:01, B*18:01~C*12:03, and B*50:01~DPB1*104:01.

**Table S2 – Demographic Characteristics of the Infection Responses ESRD Cohort**

Table S2 – Clinical characteristics of the study participants (n=327), the attribute found to be significantly associated with seroconversion was dialysis subgroup. Proportional assessment was conducted with Mann U Whitney testing (continuous data) or Chi-Squared (discrete data) testing where appropriate and reported p-values correspond with the results of these tests. (Control indicates those never infected and Case indicates those who have tested positive for COVID-19 during the study period).

**Table S3 – Cohort-Wide HLA Frequencies**

*Table S3- HLA frequencies for each allele that appeared in the cohort (n=225). HLA class I alleles A, B, and C were included along with HLA class II alleles DRB1, DQB1, and DPB1*
